# Hemostasis in Liver Disease Within Patient Blood Management: A Scoping Review of the Current Literature

**DOI:** 10.3390/jcm15093296

**Published:** 2026-04-26

**Authors:** Piotr F. Czempik, Michał Gałuszewski, Jan Olszewski, Seweryn Kaczara

**Affiliations:** 1 Department of Patient Blood Management, Department of Anesthesiology and Intensive Care, Faculty of Medical Sciences in Katowice, Medical University of Silesia, 40-752 Katowice, Poland; 2 Transfusion Committee, University Clinical Center of Medical University of Silesia in Katowice, 40-752 Katowice, Poland; 3 Student Scientific Society, Department of Anesthesiology and Intensive Care, Faculty of Medical Sciences in Katowice, Medical University of Silesia, 40-752 Katowice, Poland

**Keywords:** acute liver failure, bleeding, cirrhosis, coagulation, hemostasis, patient blood management, thrombosis, viscoelastic testing

## Abstract

**Background/Objectives**: The objective of this study was to map and synthesize the current evidence on hemostasis in chronic and acute liver disease within the framework of Patient Blood Management (PBM). **Methods**: Because research in this field is heterogeneous—spanning mechanistic studies, observational data, randomized controlled trials, guidelines, and expert reviews—a scoping review was selected to comprehensively map concepts. Findings were synthesized narratively to reflect the breadth and heterogeneity of available research. **Results**: Hemostasis in liver disease is characterized by a fragile state of rebalanced coagulation, where parallel reductions in pro- and anticoagulant factors coexist with variable fibrinolytic disturbances and thrombocytopenia. Conventional coagulation tests (CCTs) do not accurately reflect bleeding risk, whereas viscoelastic assays and thrombomodulin-modified thrombin generation testing provide a more physiologic assessment, though with limitations. Most bleeding events arise from portal hypertension rather than coagulopathy, and the routine prophylactic correction of abnormal results of CCTs is not supported by evidence. PBM-aligned strategies—such as restrictive transfusion, targeted fibrinogen replacement, and use of thrombopoietin receptor agonists (TPO-RAs)—reduce unnecessary blood product use. Thrombosis burden is increasingly recognized in this patient population. Anticoagulation is generally safe when individualized to liver function and clinical context, however significant variability persists in clinical practice, and high-quality data remain limited for advanced disease. **Conclusions**: Hemostasis in liver disease reflects a dynamic and unstable equilibrium rather than a simple bleeding tendency. Diagnostic and therapeutic strategies grounded in PBM principles improve safety by avoiding unnecessary transfusion and emphasize individualized care. Despite advances in understanding rebalanced hemostasis, major gaps remain in predicting thrombotic risk, standardizing advanced coagulation testing, and defining optimal management across disease stages.

## 1. Introduction

Hemostasis in liver disease is a complex and evolving field. Modern evidence shows that patients do not simply have a bleeding tendency but instead exist in a fragile state of rebalanced hemostasis, where reductions in pro- and anticoagulant factors, altered fibrinolysis, thrombocytopenia, and inflammation interact to create risks of both bleeding and thrombosis. Conventional coagulation tests do not reflect this physiology, and newer tools such as viscoelastic hemostatic assays (VHAs) and thrombin generation assays (TGAs) are variably used and interpreted. Clinical practice is further complicated by heterogeneous etiologies of liver disease, differing hemostatic profiles in chronic versus acute liver failure, and inconsistent transfusion and anticoagulation strategies across settings.

Because the evidence on hemostasis in liver disease is dispersed across hepatology, hematology, transfusion medicine, and critical care—and includes diverse study types, such as mechanistic research, observational studies, randomized controlled trials (RCTs), clinical guidelines, and narrative reviews—a scoping review was the most appropriate methodological approach. Unlike a systematic review, which is designed to answer narrowly defined questions about specific interventions, a scoping review enables the comprehensive mapping of broad and heterogeneous evidence, clarification of key concepts, and identification of knowledge gaps relevant to PBM. To ensure transparency and reproducibility, study selection followed the Population–Concept–Context (PCC) framework and included: adult patients with chronic or acute liver disease (population); studies addressing coagulation, fibrinolysis, laboratory assessment, bleeding or thrombosis risk, or PBM-aligned management (concept); and clinical settings in which hemostasis influences decision making (context).

This review aims to clarify what is currently known about hemostasis in liver disease and how this knowledge informs PBM.

## 2. Materials and Methods

### 2.1. Eligibility Criteria

Study selection followed the PCC framework recommended for scoping reviews. This approach ensured a comprehensive yet structured mapping of evidence relevant to hemostasis in liver disease within the context of PBM.

### 2.2. Population

The included studies involved adult patients with: chronic liver disease (CLD), cirrhosis, acute liver failure (ALF), or acute-on-chronic liver failure (ACLF). These conditions share core disturbances in hemostasis and represent the full clinical spectrum relevant to PBM. Pediatric and animal studies were excluded because their hemostatic profiles and clinical management do not generalize to adult population.

### 2.3. Concept

Eligible studies examined one or more domains of hemostasis, including: coagulation and anticoagulation pathways, fibrinolysis, laboratory assessment (CCTs, VHAs, TGAs), bleeding or thrombosis risk, periprocedural management, and PBM-aligned therapeutic strategies (e.g., transfusion thresholds, factor replacement, TPO-RAs. These domains reflect the essential components required to understand and manage hemostasis in liver disease.

### 2.4. Context

We included studies conducted in clinical settings where hemostasis influences decision making, including: inpatient care, critical care, procedural and perioperative settings. This broad context was necessary to capture the full range of PBM-related practices.

### 2.5. Study Types

Given the heterogeneity of the field, we included: observational studies, RCTs, mechanistic and laboratory research, clinical guidelines and consensus statements, narrative and expert reviews, and case reports when they contributed unique mechanistic or clinical insights. This inclusive approach aligns with the purpose of a scoping review, which aims to map the breadth of available evidence rather than restrict analysis to specific study designs.

### 2.6. Restrictions

Only English-language publications were included to ensure feasibility of screening and interpretation. The review emphasized studies from the past 15 years, reflecting the emergence of modern concepts such as rebalanced hemostasis, VHAs, and PBM. Studies unrelated to hemostasis or PBM were excluded.

### 2.7. Information Sources

The search drew on multiple sources to ensure broad coverage of the published literature on hemostasis in liver disease. Major biomedical databases (MEDLINE/PubMed, Embase, Scopus, Web of Science, Cochrane Library) were searched. The most recent search was completed on 1 March 2026 to ensure that the review reflects current developments. 

### 2.8. Search Strategy

The full search strategy was as follows: (“liver disease” OR “cirrhosis” OR “acute liver failure” OR “acute-on-chronic liver failure”) AND (“hemostasis” OR “coagulation” OR “fibrinolysis” OR “thrombosis” OR “bleeding”) AND (“viscoelastic testing” OR “TEG” OR “ROTEM” OR “thrombin generation” OR “INR” OR “fibrinogen”) AND (“patient blood management” OR “transfusion”). Reference lists of key papers were screened, and leading journals in hepatology, hematology, and transfusion medicine were hand-searched. 

## 3. Results

### 3.1. Background

Liver diseases represent a major global health burden, with substantial heterogeneity in etiology and clinical outcomes, and increasing incidence over the last decade. The global age-standardized incidence of CLD and cirrhosis currently stands at 20.7/100,000 [1]. The epidemic of CLD is anticipated to rise in parallel with diabetes and obesity [2]. The most common CLDs include metabolic dysfunction-associated steatotic liver disease (MASLD), alcohol-associated liver disease (ALD), and chronic viral hepatitis. Without timely intervention, CLDs may progress to cirrhosis, characterized by extensive fibrosis and architectural distortion [3]. Fibrosis leads to reduced hepatocellular mass and impaired liver function, accompanied by altered hepatic blood flow [4]. The clinical manifestations of cirrhosis arise both from diminished metabolic and detoxification capacities and from complications of portal hypertension (PH) [5]. Acute-on-chronic liver failure is a syndrome defined by acute decompensation of cirrhosis accompanied by failure of one or more organ systems, including the neurological, respiratory, circulatory, renal, and hemostasis systems [6], whereas acute liver failure is defined as a rapid loss of liver function in a patient without pre-existing liver disease, leading to jaundice, coagulopathy, and hepatic encephalopathy. Acute liver failure is due to viral hepatitis, drug overdose, exposure to certain toxins, and several less common causes [7].

The liver plays a central role in the synthesis and clearance of most procoagulant and anticoagulant proteins, as well as major elements of the fibrinolytic system [8]. Consequently, liver diseases are recognized as a leading cause of acquired hemostatic disturbances. Impaired synthesis of extrinsic (factor VII) and common (factors X, V, II, I) coagulation pathway factors results in a prolonged prothrombin time (PT) along with an increased international normalized ratio (INR), as well as a prolonged activated partial thromboplastin time (aPTT). These CCTs classically imply a hypocoagulable state and increased bleeding risk [9]. However, the PT assesses only the initiation phase of clot formation and therefore fails to reflect the complex hemostasis alterations observed in liver pathology. Viscoelastic hemostasis assays, e.g., thromboelastography (TEG) and rotational thromboelastometry (ROTEM), provide a more comprehensive evaluation of the platelet contribution to the clot strength and coagulation dynamics [10]. In liver diseases, VHAs support the concept of “rebalanced hemostasis” arising from parallel alterations in procoagulant and anticoagulant factors. Nevertheless, this rebalanced hemostasis is fragile, and secondary factors, such as gastrointestinal bleeding, infection, and acute kidney injury (AKI), shift the balance toward the bleeding or thrombosis phenotype. Rebalanced hemostasis in liver diseases has important consequences in the periprocedural setting and management of bleeding and thrombosis.

### 3.2. The Physiology of Hemostasis and the Role of the Liver

Hemostasis is a fundamental and dynamic physiological process essential for maintaining systemic homeostasis. Hemostasis is achieved through complex interactions involving the endothelium, platelets (PLTs), coagulation factors, natural anticoagulants, and the fibrinolytic system.

Platelets are activated by von Willebrand factor (vWF) and collagen exposed after endothelial injury, as well as by shear stress and inflammatory alterations within the endothelium, which leads to platelet adhesion [11]. Once activated, PLTs release granule contents that promote the aggregation and formation of an occlusive platelet plug. Together with vasoconstriction, platelet adhesion and aggregation constitute primary hemostasis [12]. Simultaneously, the platelet plug undergoes further modification and stabilization through incorporation of a fibrin mesh resulting from activation of the coagulation cascade. Almost all coagulation factors are synthetized in hepatocytes: fibrinogen, II, V, VII, IX, X, and XI, whereas factor VIII is synthetized in the endothelium of hepatic sinuses, and vWF in the systemic endothelium and megakaryocytes. The levels of factor VIII and vWF are increased in liver disease.

In normal conditions, coagulation factors are in balance with the natural anticoagulants antithrombin (AT), protein C (PC), and protein S (PS), maintaining an equilibrium between bleeding and thrombosis. Synthesis of natural anticoagulants also occurs in hepatocytes and is decreased in liver disease.

The main fibrinolytic protein, plasminogen, is also synthesized in hepatocytes. After activation, plasminogen is converted to plasmin, which is responsible for fibrin degradation. At the same time, the liver produces α2-antiplasmin (main plasmin inhibitor) and thrombin-activatable fibrinolysis inhibitor (TAFI). The liver is also responsible for clearance of fibrinolytic factors. Impaired hepatic clearance increases circulating tissue plasminogen activator (t-PA), which promotes plasmin generation and may tilt the system toward hyperfibrinolysis with bleeding in soft tissues following even a minor trauma [13]. However, some patients show reduced fibrinolytic capacity, which may increase thrombotic risk, leading to portal vein thrombosis (PVT). Many patients with advanced liver disease show elevated plasminogen activator inhibitor-1 (PAI-1) levels, especially during inflammatory states and in the course of ALF. Activation of plasminogen is reduced because PAI-1 profoundly inhibits t-PA.

The liver is also the primary controller of thrombopoiesis through synthesis of thrombopoietin (TPO). The normal spleen normally holds about one-third of circulating PLTs as a reserve, whereas PH leads to the splenic sequestration of PLTs. In cirrhosis, scarring increases portal pressure, which causes splenomegaly and, as a result, hypersplenism, in which more PLTs are trapped and destroyed in the spleen. Alcohol and other toxins may also depress the bone marrow production of PLTs. Platelets may also be destroyed by autoantibodies. All these mechanisms lead to thrombocytopenia. Although previous studies suggested that PLT function in liver disease may be impaired [14], limitations of the PLT function testing methodology have been recognized, and the current suggestion is that PLT function in liver disease is intact or even enhanced [15]. In liver disease, thrombocytopenia may be partially offset by increased vWF and decreased a disintegrin and metalloprotease with thrombospondin-13 domain (ADAMTS-13), which cleaves ultra-large vWF multimers circulating in the blood. These ultra-large vWF multimers are extremely prothrombotic and can cause PLTs to clump excessively. By trimming vWF, ADAMTS-13 prevents the uncontrolled PLT adhesion and formation of microthrombi in small vessels.

### 3.3. Rebalanced Hemostasis in Liver Disease: The Modern Conceptual Framework

**Figure 1:**
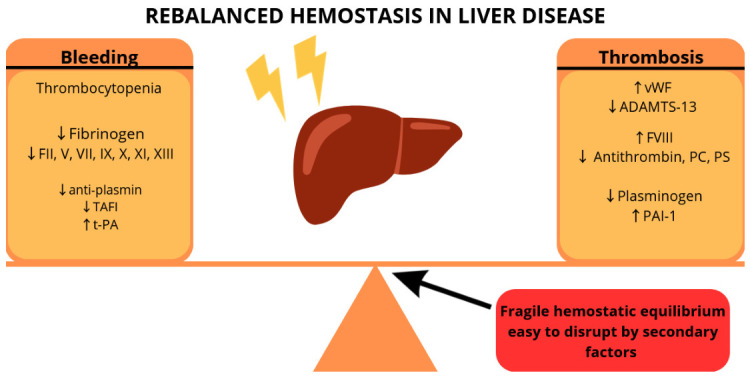
Graphical presentation of factors associated with rebalanced hemostasis in liver disease. ADAMTS-13—a disintegrin and metalloprotease with thrombospondin-13 domain; PAI-1—plasminogen activator inhibitor-1; PC—protein C; PS—protein S; TAFI—thrombin-activatable fibrinolysis inhibitor; t-PA—tissue plasminogen activator; vWF—von Willebrand factor.

Our understanding of hemostasis in liver diseases has moved from the historical “auto-anticoagulation” model to the “rebalanced hemostasis” model, in which hemostasis is characterized by parallel alterations in both procoagulant and anticoagulant factors. It is worth mentioning that factor VIII and vWF are increased in both CLD and ALF. Nevertheless, this rebalanced state is fragile, and secondary factors, such as gastrointestinal bleeding, infection, and AKI, shift the balance toward bleeding or thrombosis. Mechanisms of rebalanced hemostasis in liver disease are illustrated in Figure 1.

### 3.4. Hemostasis in Chronic Liver Disease and Cirrhosis

Patients with CLD show a gradual reduction in both procoagulant and anticoagulant proteins, vitamin K deficiency, variable fibrinolytic abnormalities, and thrombocytopenia. Despite abnormal CCTs, many patients maintain a rebalanced but frequently unstable hemostatic state, with potential for either hypocoagulability or hypercoagulability depending on triggers. Thrombocytopenia is common due to splenic sequestration and reduced TPO production. In patients with CLD due to hepatitis C, an important factor may be autoantibody-mediated PLT destruction [16]. In these patients, PLT-associated and anti-PLT antibodies are often detectable at high levels [17]. It is hypothesized that PLTs upon exposure to high shear stress and subsequent activation in PH are rapidly eliminated from the circulation. The prevalence of thrombocytopenia in CLD ranges between 15 and 75% [16]. A progressive decrease in PLTs is considered a noninvasive marker for the development of PH due to fibrosis and cirrhosis. Thrombocytopenia is not associated with spontaneous bleeding unless PLTs decrease to <50 × 10^9^ L^−1^ [18], and even then, there is no clear correlation between low PLT counts and the incidence of periprocedural bleeding [19]. Thrombocytopenia is compensated by increased vWF and reduced ADAMTS-13. The rebalanced hemostasis in CLD is usually more stable than in ALF.

### 3.5. Hemostasis in Acute Liver Failure

The etiology of ALF may be caused by several factors: acute viral hepatitis, drug-induced liver injury (e.g., acetaminophen toxicity), ischemic hepatitis (shock liver), autoimmune hepatitis flare, acute fatty liver of pregnancy, toxin exposure, and severe systemic infections. Acute liver failure produces a profoundly unstable but rebalanced hemostatic state. Patients may appear coagulopathic (very high INR) but often maintain near-normal thrombin generation. The bleeding risk is lower than the INR suggests, while the thrombotic risk is real, especially in the microcirculation. Patients are not usually thrombocytopenic because PH does not develop acutely. Most ALF patients are prothrombotic, although some may have hyperfibrinolysis. A comparison of ROTEM results in CLD and ALF is presented in Table 1.

These parameters provide clinically meaningful insight into the hemostatic profile of each condition. The clotting time (CT), which reflects the initiation phase of coagulation and depends primarily on coagulation factor levels, is typically normal or only mildly prolonged in both CLD and ALF, despite markedly abnormal CCTs. The clot formation time (CFT), representing the speed of clot propagation and influenced by fibrinogen levels and platelet function, is often prolonged in CLD due to reduced fibrinogen synthesis and thrombocytopenia, whereas it tends to remain normal in ALF. The maximum clot firmness (MCF), a measure of the overall clot strength determined by the fibrinogen and platelet contribution, is frequently decreased in CLD but preserved or even increased in ALF because of elevated factor VIII and vWF levels. Maximum lysis (ML), reflecting fibrinolytic activity, is variable in CLD—ranging from hyperfibrinolysis to fibrinolytic shutdown—while generally remaining within normal limits in ALF. These differences illustrate the relative stability of rebalanced hemostasis in CLD compared with the more dynamic and unstable hemostatic state observed in ALF.

### 3.6. Laboratory Assessment of Hemostasis in Liver Disease

The typical results of CCTs in liver disease are thrombocytopenia, a prolonged PT, a prolonged aPTT, and low fibrinogen.

Both the PT and aPTT in patients with liver disease are misleading. Interpreting these tests, one must know the methodology behind them. Both tests were originally developed to screen for hemophilias and other rare bleeding disorders. These tests are sensitive to particular factor deficiencies but not to levels of natural anticoagulants. As in liver diseases, both coagulation and anticoagulation factors are low; thus, these tests should not be used to assess hemostasis. In liver disease, hypersialylation of fibrinogen slows fibrin polymerization. This post-translational modification causes the Clauss assay to underestimate fibrinogen levels and contributes to prolongation of the TT. The final fibrin clot in liver disease has decreased permeability and is more resistant to fibrinolysis [20].

Viscoelastic hemostatic assays have been used to assess complex hemostasis in liver disease patients. These tests are point-of-care whole blood tests that assess initiation, propagation, firmness and fibrinolysis of the clot. These tests have been shown to be normal or only mildly deranged in liver disease patients despite large derangements in CCTs [21,22]. Although VHAs compared to CCTs more precisely assess hemostasis in liver disease patients, they are not without limitations. The potential drawback is the inability of these tests to assess the PC system, which requires endothelial transmembrane protein thrombomodulin (TM) for activation; vWF, which requires shear stress for activation; and the different architecture of the fibrin clot, which is less permeable for fibrinolytic proteins [22]. Table 1 summarizes the characteristic differences in the ROTEM parameters between CLD and ALF.

The test that could be useful for assessment of complex hemostasis in patients with liver disease is the TGA that uses TM and is therefore sensitive to both coagulation and anticoagulation factors. This test has been shown to be normal in patients with CLD, normal or elevated in patients with ALF or ACLF [22].

Platelet count does not give information on platelet functionality in vivo. The rebalanced hemostasis model indicates that thrombocytopenia in liver disease is compensated for by increased levels of vWF and decreased levels of ADAMTS-13.

To sum up, CCTs do not reflect hemostasis in liver disease. Much more useful are VHAs, although they are not sensitive to PC, vWF, or the changed architecture of the fibrin clot. The most accurate test is the TM-modified TGA (TM-TGA); however, it is not widely available and remains a research tool.

### 3.7. Hemostatic Implications for Invasive Procedures and Surgery in Patients with Liver Disease in the Context of Patient Blood Management

Although bleeding risk in liver disease has traditionally been attributed to “coagulopathy,” contemporary evidence demonstrates substantial variability across studies. Several observational cohorts report low rates of procedure-related bleeding even in the presence of markedly abnormal CCTs, supporting the concept of rebalanced hemostasis. However, other studies—particularly those involving patients with acute decompensation, infection, or AKI—describe higher bleeding rates, highlighting that secondary clinical stressors can destabilize the hemostatic balance. Meta-analytic data further illustrate this heterogeneity: pooled analyses of paracentesis and thoracentesis show major bleeding rates below 1%, whereas endoscopic and surgical procedures demonstrate wider variability depending on the disease severity and procedural complexity [23,24,25]. These discrepancies underscore the importance of individualized risk assessment rather than reliance on CCT thresholds alone.

Hemorrhagic complications following invasive procedures are less common than previously presumed. Bleeding in cirrhotic patients admitted to the intensive care unit (ICU) is relatively rare (14%) and typically reflects advanced CLD, higher Model of End-Stage Liver Disease (MELD) scores, and severe ACLF [26]. Most bleeding events are not due to hemostasis failure but PH (variceal bleeding) or mechanical causes [27]. Approximately 90% of bleeding events are attributable to PH. Bleeding complications are also uncommon in ALF and rarely require specific intervention despite the presence of markedly abnormal hemostatic profiles in the most severe ALF phenotypes [28]. Profound vWF/ADAMTS-13 imbalance, rather than impaired coagulation or fibrinolysis alone, correlated with poorer ALF outcomes and increased bleeding events. Most bleeding episodes in this population were upper gastrointestinal bleeds, attributed not to coagulopathy but to “stress-related mucosal disease,” an inflammatory manifestation of critical illness [29].

In hospitalized patients with acutely decompensated liver disease, TM-TGA may predict major bleeding following invasive procedures, particularly when the pre-procedural endogenous thrombin potential (ETP) is <350 nmol L^−1^ min^−1^ [30]. Procedure-related bleeding is more frequent in patients with MASLD, elevated MELD score, AKI, infection, and higher body mass index (BMI) [31]. A fourfold increase in bleeding risk in decompensated cirrhotic patients with AKI has been shown, recommending management of renal dysfunction prior to invasive procedures to limit bleeding-related complications [32].

Low-risk procedures (paracentesis, thoracentesis, diagnostic endoscopy) have very low major bleeding rates, even with abnormal CCTs; therefore, routine correction is not recommended before low-risk procedures in stable cirrhosis by major societies (AGA, AASLD, ASGE). Higher-risk procedures (percutaneous liver biopsy, complex therapeutic endoscopy) warrant more individualized assessment and, often, alternative approaches (e.g., transjugular biopsy). Use of blood products or factor concentrates is considered pre-procedure mainly when there is active bleeding or disseminated intravascular coagulation (DIC), PLTs are extremely low (<10 × 10^9^ L^−1^), or there is profound hypofibrinogenemia, as well as in higher-risk procedures (percutaneous biopsy, large endoscopic mucosal resection/endoscopic submucosal dissection, complex endoscopic retrograde cholangiopancreatography) when safer alternatives are not feasible.

Viscoelastic testing reduces unnecessary transfusions in cirrhosis without increasing bleeding risk, especially before low-risk, non-surgical procedures. Evidence supports VHA-guided algorithms as safer and more efficient than INR/platelet-based transfusion strategies [33,34].

Anticoagulation can be safely used in cirrhosis, but the choice of agent depends heavily on the Child–Pugh class, renal function, and indication. Direct oral anticoagulants (DOACs), especially apixaban, appear at least as safe—and often safer—than warfarin in Child–Pugh A–B, while low-molecular-weight heparin (LMWH) remains preferred in advanced (Child–Pugh C) disease [35].

### 3.8. Prevention and Treatment of Bleeding in Patients with Liver Disease in the Context of Patient Blood Management 

Blood components such as fresh frozen plasma (FFP) and platelet concentrate (PC), are widely used for the prevention and treatment of bleeding in CLD and ALF. Clinicians frequently attempt to correct markedly abnormal CCT values by administering transfusions [27,36]. However, according to the most recent guidelines from the European Society of Anesthesiology and Intensive Care, the routine prophylactic correction of CCT results using blood products in CLD and ALF is not recommended [37]. Similarly, the European Association for the Study of the Liver states that in patients with cirrhosis undergoing invasive procedures, correction of a prolonged INR with FFP does not reduce clinically relevant procedure-related bleeding [38]. This reflects both the potential for adverse effects associated with transfusions and the limited evidence supporting their clinical effectiveness [38,39]. Clinical outcomes further reinforce these concerns. Attempts to normalize misleadingly elevated INR values in non-bleeding patients with liver disease may also predispose them to thromboembolic events [40]. In the study by von Meijenfeldt et al., administration of FFP and PC resulted in a prothrombotic state in nearly all patients [41]. Biswas et al. reported that FFP and PC transfusions did not improve hemostasis in cirrhotic patients with acute variceal bleeding and were associated with increased re-bleeding at 5 and 42 days [10]. Mohanty et al. found a 37% vs. 6.9% mortality rate in transfused patients, reduced bleeding control efficacy, and prolonged hospitalization [42]. Fu et al. demonstrated that FFP transfusions were associated with increased risks of liver, coagulation, and respiratory failure, with higher transfusion volumes correlating with poorer outcomes in decompensated cirrhosis [43].

Alternative strategies have therefore been explored. Hartman et al. reported that liver transplantation can be performed safely without FFP when guided by the ROTEM-directed administration of coagulation factor concentrates, concluding that factor concentrates were superior to FFP [44]. In their analysis, PCs, blood loss, and MELD scores, but not fibrinogen or prothrombin complex concentrate (PCC), were independent predictors of mortality [44]. Conversely, Stravitz et al. proposed a hierarchy of blood components in ALF, identifying PC as the most beneficial, while emphasizing that FFP does not provide clinical benefit [28]. Overall, individualized approach is required, as highlighted by Janko et al., who reported significant heterogeneity in transfusion practices and persistent discrepancies between guideline recommendations and real-world management [36].

Patients with liver disease may present with vitamin K deficiency, which impairs γ-carboxylation of several clotting factors (II, VII, IX, X) and natural anticoagulants (proteins C and S). The risk factors for vitamin K deficiency in liver disease are as follows: cholestasis, malnutrition, prolonged antibiotic use, and a long ICU stay. Vitamin K in liver disease is administered intravenously due to reduced absorption from the gut. Care must be taken during parenteral administration of vitamin K, as anaphylactic reactions occasionally occur.

A relatively new therapeutic option for thrombocytopenia in liver disease are TPO-RAs, such as avatrombopag and lusutrombopag, which offer a non-transfusion approach to increasing PLTs in patients scheduled for invasive procedures. Avatrombopag was studied in two identically designed RCTs (ADAPT-1 and ADAPT-2) in adult patients with thrombocytopenia and CLD undergoing an elective procedure. The doses used were 60 mg (baseline PLTs < 40 × 10^9^ L^−1^) or 40 mg once daily (baseline PLT: 40–49 × 10^9^ L^−1^) for 5 days, and the invasive procedure was performed 10–13 days after randomization. The mean PLT change in subjects with lower baseline PLTs was 31.3–32.0 × 10^9^ L^−1^, whereas in subjects with higher baseline PLTs 37.1–44.9 × 10^9^ L^−1^. Administration of avatrombopag approximately 10 days prior to the procedure is required to achieve therapeutic effect [45]. Lusutrombopag was studied in an RCT with doses of 3 mg given once daily for 7 days. The median increase in PLTs was 45.0 × 10^9^ L^−1^ [16]. Furuichi et al. demonstrated the longer persistence of platelet elevation and a greater increase in platelet counts following TPO-RA therapy compared with platelet transfusion, with treatment response influenced by factors such as low hemoglobin and splenomegaly [46]. These agents effectively increase PLTs, reduce transfusion requirements, and decrease the risk of periprocedural bleeding. Eguchi et al. reported that platelet counts did not exceed 200 × 10^9^ L^−1^ under avatrombopag therapy, suggesting mitigation of thromboembolic risk [47], while Poordad et al. confirmed the safety and tolerability of tailored dosing [48]. Severe thrombocytopenia (<30 × 10^9^ L^−1^) predicts treatment failure and represents a contraindication to TPO-RA use [49]. Xiao et al. noted that clinical implementation remains limited, potentially due to the treatment cost and the required preoperative waiting period, although these agents appear particularly beneficial for patients undergoing planned or repeated procedures [50].

Cryoprecipitate (CRYO) is a plasma-derived product enriched in fibrinogen, FVIII, FXIII, vWF, and fibronectin. Patients with CLD or ALF may develop hypofibrinogenemia, especially during acute decompensation, infection, or massive transfusion. Cryoprecipitate is often used when fibrinogen levels are significantly reduced, bleeding is ongoing, and there is no fibrinogen concentrate available. This aligns with PBM principles that prioritize targeted factor replacement over FFP transfusion. However, CRYO has several drawbacks: variable fibrinogen contents, the requirement for transfusion compatible within the AB0 group, and the risk of transfusion-related complications.

### 3.9. Therapeutic Considerations for Thrombosis in Patients with Liver Disease

The literature on thrombosis risk in liver disease is inconsistent. While several large cohort studies demonstrate increased rates of PVT and venous thromboembolism (VTE) in cirrhosis, other studies report no significant difference compared with non-cirrhotic controls after adjusting for confounders. Differences in diagnostic criteria, imaging frequencies, and patient selection likely contribute to these conflicting findings. Meta-analyses suggest an overall increased risk of PVT in cirrhosis, but with substantial heterogeneity and wide confidence intervals [51,52]. Studies evaluating VHAs also show mixed results: some identify hypercoagulable profiles associated with thrombosis, whereas others report normal or even hypocoagulable patterns. These inconsistencies reflect the complex and dynamic nature of hemostasis in liver disease and the limitations of the current diagnostic tools.

The majority of patients with liver diseases exhibit an increased risk of venous thrombosis, particularly PVT, with thrombotic events occurring more frequently following acute liver decompensation. Other thrombotic complications, including VTE, such as deep vein thrombosis (DVT), pulmonary embolism (PE), and ischemic stroke, have also been reported in patients with liver cirrhosis [53]. The highest prevalence of early VTE is observed in patients with MASLD, whereas the lowest prevalence in those with ALF [26].

The prothrombotic state observed in patients with liver disease is partly mediated by systemic inflammation. The severity of inflammation is greater in individuals who develop thrombosis, even though the coagulation and fibrinolytic parameters are largely comparable between groups, with the notable exception of elevated PAI-1 in patients who developed thrombosis. The authors hypothesize that a baseline PAI-1 > 50 ng mL^−1^ can serve as a predictor of VTE [54]. In addition, inflammatory states are associated with increased neutrophil counts and the release of neutrophil extracellular traps (NETs), which can promote thrombus formation. Accumulation of NET-producing neutrophils within the injured liver may contribute to disease progression, while interactions between neutrophils and PLTs further promote PLT activation and deposition [41]. Importantly, systemic inflammation may predispose patients to both thrombotic and bleeding complications, but findings across studies remain inconsistent, underscoring the need for further investigation [54].

Increased TG, which may further augment thrombotic risk, has been observed in patients with CLD, ALF, and sepsis. Although the mechanisms underlying this phenomenon remain unclear and do not appear to involve enhanced intrinsic or extrinsic pathway activation, it is suggested that the low-grade activation of coagulation associated with deficiencies in natural anticoagulants may play a contributory role [55].

Beyond systemic events, a prothrombotic state within the microvasculature of the injured liver and peripheral tissues may contribute to progression of the primary liver disease [56]. Microthrombus formation may result in multiple organ system failure (MOSF) secondary to hypoxic injury [57]. Reduced activity of ADAMTS-13 in liver disease promotes a prothrombotic intrahepatic environment by allowing for accumulation of large vWF multimers. When ADAMTS-13 is low, plasmin may instead cleave vWF into cleaved vWF (cvWF), which may represent an adaptive response to microvascular thrombosis. Marked ADAMTS-13 reduction has been reported in ACLF, ALF, and sepsis, correlating with disease severity, and cvWF may serve as a marker of progression, though its clinical value remains uncertain [58,59].

A clinically important thrombophilic condition in CLD is the presence of antiphospholipid antibodies (aPLs). These antibodies may appear as false positives due to hepatic dysfunction, infection, or polyclonal activation, but true antiphospholipid syndrome (APS) can occur and is associated with PVT, graft complications after liver transplantation, and difficult anticoagulation decisions. Careful laboratory confirmation and clinical correlation are therefore essential to distinguish incidental aPL positivity from a genuine prothrombotic APS. This issue is particularly relevant in autoimmune hepatitis, where overlapping autoimmune features increase the likelihood of true APS [60].

Low serum albumin, a hallmark of advanced cirrhosis, has recently been shown to promote PLT activation and is independently associated with PVT, suggesting that albumin functions as a modulator of the hemostatic system rather than as merely a marker of liver dysfunction [61].

Portal vein thrombosis is a frequent complication of cirrhosis, and prior PVT has been shown to be an independent predictor of both PVT recurrence and reduced survival, underscoring its prognostic significance [62].

Clinically, PVT worsens outcomes. Complete PVT occlusion significantly increases 6-week mortality after acute variceal bleeding, while early transjugular intrahepatic portosystemic shunt (TIPS) improves survival [63]. PVT also increases upper GI bleeding risk by worsening PH, and thrombotic events often precede bleeding [64]. Management options include thrombectomy, thrombolysis, TIPS, and anticoagulation [65]. Despite historical concerns about bleeding, guidelines now support LMWHs, vitamin K antagonists (VKAs), and DOACs in liver disease. Direct oral anticoagulants are at least as effective as LMWHs and VKAs for PVT recanalization, with similar or lower bleeding risk [66]. Rivaroxaban and dabigatran are effective without increasing bleeding [67], and rivaroxaban may reduce PH complications and improve survival [53]. Anticoagulation overall reduces mortality, complications, and hospital stay [62]. Direct oral anticoagulants may offer advantages over VKAs, including better safety and shorter length of stay [68,69].

Overall, despite multiple therapeutic options, optimal thrombosis management in liver disease remains uncertain, especially in advanced cirrhosis, as most studies exclude high-risk patients. Therefore more robust prospective data are needed.

## 4. Conclusions

Hemostasis in liver disease reflects a delicate and dynamically shifting balance in which bleeding and thrombosis risks coexist and change with clinical stressors. Conventional coagulation tests do not capture this complexity, whereas VHAs and TGAs provide more physiologically relevant—though still imperfect—insights into global hemostatic function. Current evidence supports a PBM-aligned approach that avoids reflexive correction of laboratory abnormalities, emphasizes procedure-specific risk assessment, and uses targeted interventions such as TPO-RAs, fibrinogen replacement, or anticoagulation. In the complex hemostasis scenario of liver disease at a particular level of dysfunction, caused by a particular etiology, with certain exacerbating factors, personalized hemostasis management is of great importance. 

## 5. Future Directions and Research Gaps

Overall, the heterogeneity and methodological limitations of the evidence base reinforce the importance of individualized, physiology-based approach to hemostasis assessment and PBM-aligned management in liver disease. Further research is needed to address persistent gaps, including standardized VHA protocols, validated thrombotic risk prediction tools, and targeted hemostatic interventions. Although VHAs are better than CCTs at assessing complex hemostasis in liver disease, they are not without limitations. Therefore, there is a need to improve their accuracy in liver disease, or to introduce research methodologies (TM-TGA) into clinical practice. Another unresolved issue is the standardization of reference ranges for VHAs in CLD. The task is difficult because many different etiologies lead to liver disease. It may be possible that different etiologies have different reference ranges. The major challenge recently is understanding thrombotic risk in compensated vs. decompensated cirrhosis and managing thrombotic risk in these patients. There are also emerging developments in pharmaceutical research focused on novel therapeutics targeting fibrinolysis and PLT function.

## Figures and Tables

**Table 1:** Comparison of rotational thromboelastometry parameters in chronic and acute liver disease.

Parameter	Chronic Liver Disease	Acute Liver Disease
INTEM ^1^ CT ^2^	Normal or mildly prolonged	Mildly prolonged
EXTEM ^3^ CT	Normal of mildly prolonged	Normal or mildly prolonged
EXTEM CFT ^4^	Prolonged	Normal
EXTEM MCF ^5^	Decreased	Normal or increased
EXTEM ML ^6^	Normal/decreased/increased	Normal
FIBTEM ^7^ MCF	Decreased	Normal initially

^1^ Intrinsic coagulation pathway assay; ^2^ clotting time; ^3^ extrinsic coagulation pathway assay; ^4^ clot formation time; ^5^ maximal clot firmness; ^6^ maximal lysis; ^7^ fibrinogen assay.

## Data Availability

No new data were created or analyzed in this study. Data sharing is not applicable to this article.

## References

[1] Moon A.M., Singal A.G., Tapper E.B. (2020). Contemporary Epidemiology of Chronic Liver Disease and Cirrhosis. Clin. Gastroenterol. Hepatol..

[2] Cheemerla S., Balakrishnan M. (2021). Global Epidemiology of Chronic Liver Disease. Clin. Liver Dis..

[3] Manikat R., Ahmed A., Kim D. (2024). Current epidemiology of chronic liver disease. Gastroenterol. Rep..

[4] Guixé-Muntet S., Quesada-Vázquez S., Gracia-Sancho J. (2024). Pathophysiology and therapeutic options for cirrhotic portal hypertension. Lancet Gastroenterol. Hepatol..

[5] Bernal W., Jalan R., Quaglia A., Simpson K., Wendon J., Burroughs A. (2015). Acute-on-chronic liver failure. Lancet.

[6] Shingina A., Mukhtar N., Wakim-Fleming J., Alqahtani S., Wong R.J., Limketkai B.N., Larson A.M., Grant L. (2023). Acute Liver Failure Guidelines. Am. J. Gastroenterol..

[7] Maiwall R., Kulkarni A.V., Arab J.P., Piano S. (2024). Acute liver failure. Lancet.

[8] Lu L., Zhu C., Zhou D., Li S., Yi L., Wei S., Peng Q. (2024). Interaction between coagulation and inflammatory system in liver disease: Re-focus on hematological markers. Biomark. Med..

[9] Fierro-Angulo O.M., González-Regueiro J.A., Pereira-García A., Ruiz-Margáin A., Solis-Huerta F., Macías-Rodríguez R.U. (2024). Hematological abnormalities in liver cirrhosis. World J. Hepatol..

[10] Biswas S., Vaishnav M., Pathak P., Gunjan D., Mahapatra S.J., Kedia S., Rout G., Thakur B., Nayak B., Kumar R. (2022). Effect of thrombocytopenia and platelet transfusion on outcomes of acute variceal bleeding in patients with chronic liver disease. World J. Hepatol..

[11] van Dievoet M.A., Eeckhoudt S., Stephenne X. (2020). Primary Hemostasis in Chronic Liver Disease and Cirrhosis: What Did We Learn over the Past Decade?. Int. J. Mol. Sci..

[12] Zanetto A., Campello E., Senzolo M., Simioni P. (2024). The evolving knowledge on primary hemostasis in patients with cirrhosis: A comprehensive review. Hepatology.

[13] Hrudya, Mallikarjun, Muddala D., Siddarth G.R., Godha H., Kurien S.S., Devarbhavi H. (2025). Hyperfibrinolysis: An Underrecognized Cause of Bleeding in Cirrhosis. J. Clin. Exp. Hepatol..

[14] Lee M.Y., Verni C.C., Herbig B.A., Diamond S.L. (2017). Soluble fibrin causes an acquired platelet glycoprotein VI signaling defect: Implications for coagulopathy. J. Thromb. Haemost..

[15] Raparelli V., Basili S., Carnevale R., Napoleone L., Del Ben M., Nocella C., Bartimoccia S., Lucidi C., Talerico G., Riggio O. (2017). Low-grade endotoxemia and platelet activation in cirrhosis. Hepatology.

[16] Peck-Radosavljevic M., Simon K., Iacobellis A., Hassanein T., Kayali Z., Tran A., Makara M., Ben Ari Z., Braun M., Mitrut P. (2019). Lusutrombopag for the Treatment of Thrombocytopenia in Patients with Chronic Liver Disease Undergoing Invasive Procedures (L-PLUS 2). Hepatology.

[17] Pradella P., Bonetto S., Turchetto S., Uxa L., Comar C., Zorat F., De Angelis V., Pozzato G. (2011). Platelet production and destruction in liver cirrhosis. J. Hepatol..

[18] Tripodi A., Primignani M., Chantarangkul V., Mannucci P.M. (2010). More on: Enhanced thrombin generation in patients with cirrhosis-induced coagulopathy. J. Thromb. Haemost..

[19] Brown R.S. (2019). Current Management of thrombocytopenia in chronic liver disease. Gastroenterol. Hepatol..

[20] Hugenholtz G.C.G., Macrae F., Adelmeijer J., Dulfer S., Porte R.J., Lisman T., Ariëns R.A.S. (2016). Procoagulant changes in fibrin clot structure in patients with cirrhosis are associated with oxidative modifications of fibrinogen. J. Thromb. Haemost..

[21] Raeven P., Baron-Stefaniak J., Simbrunner B., Stadlmann A., Schwabl P., Scheiner B., Schaden E., Eigenbauer E., Quehenberger P., Mandorfer M. (2020). Thromboelastometry in patients with advanced chronic liver disease stratified by severity of portal hypertension. Hepatol. Int..

[22] Lisman T., Arefaine B., Adelmeijer J., Zamalloa A., Corcoran E., Smith J.G., Bernal W., Patel V.C. (2020). Global hemostatic status in patients with acute-on-chronic liver failure and septics without underlying liver disease. J. Thromb. Haemost..

[23] Fong C., Tan C.W.C., Tan D.K.Y., See K.C. (2021). Safety of thoracentesis and tube thoracostomy in patients with uncorrected coagulopathy: A Systematic review and meta-analysis. Chest.

[24] Park S.B., Jeon J.W., Shin H.P. (2023). The Risk of endoscopy-related bleeding in patients with liver cirrhosis: A retrospective study. Medicina.

[25] Newman K.L., Johnson K.M., Cornia P.B., Wu P., Itani K., Ioannou G.N. (2020). Perioperative Evaluation and Management of Patients with Cirrhosis: Risk Assessment, Surgical Outcomes, and Future Directions. Clin. Gastroenterol. Hepatol..

[26] Ow T.W., Fatourou E., Rabinowich L., van den Boom B., Nair S., Patel V.C., Hogan B., McPhail M., Roberts L.N., Bernal W. (2022). Prevalence of Bleeding and Thrombosis in Critically Ill Patients with Chronic Liver Disease. Thromb. Haemost..

[27] Northup P.G., Lisman T., Roberts L.N. (2021). Treatment of bleeding in patients with liver disease. J. Thromb. Haemost..

[28] Stravitz R.T., Fontana R.J., Meinzer C., Durkalski-Mauldin V., Hanje A.J., Olson J., Koch D., Hamid B., Schilsky M.L., McGuire B. (2021). Coagulopathy, Bleeding Events, and Outcome According to Rotational Thromboelastometry in Patients with Acute Liver Injury/Failure. Hepatology.

[29] Driever E.G., Stravitz R.T., Zhang J., Adelmeijer J., Durkalski V., Lee W.M., Lisman T. (2021). VWF/ADAMTS13 Imbalance, But Not Global Coagulation or Fibrinolysis, Is Associated with Outcome and Bleeding in Acute Liver Failure. Hepatology.

[30] Zanetto A., Campello E., Bulato C., Willems R., Konings J., Roest M., Gavasso S., Nuozzi G., Toffanin S., Burra P. (2025). Impaired whole blood thrombin generation is associated with procedure-related bleeding in acutely decompensated cirrhosis. J. Hepatol..

[31] Intagliata N.M., Rahimi R.S., Higuera-de-la-Tijera F., Simonetto D.A., Farias A.Q., Mazo D.F., Boike J.R., Stine J.G., Serper M., Pereira G. (2023). Procedural-Related Bleeding in Hospitalized Patients with Liver Disease (PROC-BLeeD): An International, Prospective, Multicenter Observational Study. Gastroenterology.

[32] Hung A., Garcia-Tsao G. (2018). Acute kidney injury, but not sepsis, is associated with higher procedure-related bleeding in patients with decompensated cirrhosis. Liver Int..

[33] Shenoy A., Louissaint J., Shannon C., Tapper E.B., Lok A.S. (2022). Viscoelastic Testing Prior to Non-surgical Procedures Reduces Blood Product Use Without Increasing Bleeding Risk in Cirrhosis. Dig. Dis. Sci..

[34] Kumar R., Ng L.X.L., Wong Y.J., Tan C.K., Wang L.Z., Qiu T.Y., Wong B., Lin K.W., Li J.W., Kwek A.B. (2025). Rotational Thromboelastometry Reduces the Need for Preemptive Transfusion in Cirrhosis: A Randomized Controlled Trial (NCT:05698134). J. Clin. Exp. Hepatol..

[35] Pereira Portela C., Gautier L.A., Zermatten M.G., Fraga M., Moradpour D., Bertaggia Calderara D., Aliotta A., Veuthey L., De Gottardi A., Stirnimann G. (2024). Direct oral anticoagulants in cirrhosis: Rationale and current evidence. JHEP Rep..

[36] Janko N., Majeed A., Clements W., Fink M.A., Lubel J., Goodwin M., Nicoll A., Strasser S.I., Sood S., Bollipo S. (2023). Wide variation in pre-procedural blood product transfusion practices in cirrhosis: A national multidisciplinary survey. Hepatol. Commun..

[37] Kietaibl S., Ahmed A., Afshari A., Albaladejo P., Aldecoa C., Barauskas G., De Robertis E., Faraoni D., Filipescu D.C., Fries D. (2023). Management of severe peri-operative bleeding: Guidelines from the European Society of Anaesthesiology and Intensive Care: Second update 2022. Eur. J. Anaesthesiol..

[38] European Association for the Study of the Liver (2022). EASL Clinical Practice Guidelines on prevention and management of bleeding and thrombosis in patients with cirrhosis. J. Hepatol..

[39] Rassi A.B., d’Amico E.A., Tripodi A., da Rocha T.R.F., Migita B.Y., Ferreira C.M., Carrilho F.J., Farias A.Q. (2020). Fresh frozen plasma transfusion in patients with cirrhosis and coagulopathy: Effect on conventional coagulation tests and thrombomodulin-modified thrombin generation. J. Hepatol..

[40] Czempik P.F., Płonka J. (2024). No bleeding complications during major surgery in a subacute liver dysfunction patient with international normalized ratio in the therapeutic range. Acta Haematol. Pol..

[41] von Meijenfeldt F.A., Lisman T., Pacheco A., Zen Y., Bernal W. (2025). Histologic evidence of neutrophil extracellular traps and fibrin(ogen) deposition in liver biopsies from patients with inflammatory liver disease. Res. Pract. Thromb. Haemost..

[42] Mohanty A., Kapuria D., Canakis A., Lin H., Amat M.J., Rangel Paniz G., Placone N.T., Thomasson R., Roy H., Chak E. (2021). Fresh frozen plasma transfusion in acute variceal haemorrhage: Results from a multicentre cohort study. Liver Int. Off. J. Int. Assoc. Study Liver.

[43] Fu X., Yan D., Huang W., Xie X., Zhou Y., Li H., Wang Y., Pei S., Yao R., Li N. (2024). Efficacy of fresh frozen plasma transfusion in decompensated cirrhosis patients with coagulopathy admitted to ICU: A retrospective cohort study from MIMIC-IV database. Sci. Rep..

[44] Hartmann M., Walde C., Dirkmann D., Saner F.H. (2019). Safety of coagulation factor concentrates guided by ROTEMTM-analyses in liver transplantation: Results from 372 procedures. BMC Anesthesiol..

[45] Tanaka K., Baba T., Yoshida M., Iguchi M., Sonoyama T., Fukuhara T., Kano T. (2022). Relationship between baseline clinical characteristics and efficacy of lusutrombopag in thrombocytopenic patients with chronic liver disease: Post hoc analysis of two placebo-controlled phase 3 trials. Curr. Med. Res. Opin..

[46] Furuichi Y., Takeuchi H., Yoshimasu Y., Kasai Y., Abe M., Itoi T. (2020). Thrombopoietin receptor agonist is more effective than platelet transfusion for chronic liver disease with thrombocytopenia, shown by propensity score matching. Hepatol. Res..

[47] Eguchi Y., Takahashi H., Mappa S., Santagostino E. (2022). Phase 2 study of avatrombopag in Japanese patients with chronic liver disease and thrombocytopenia. Hepatol. Res..

[48] Poordad F., Terrault N.A., Alkhouri N., Tian W., Allen L.F., Rabinovitz M. (2020). Avatrombopag, an Alternate Treatment Option to Reduce Platelet Transfusions in Patients with Thrombocytopenia and Chronic Liver Disease-Integrated Analyses of 2 Phase 3 Studies. Int. J. Hepatol..

[49] Hirooka M., Ochi H., Hiraoka A., Koizumi Y., Tanaka T., Sunago K., Yukimoto A., Imai Y., Watanabe T., Yoshida O. (2020). Role of severe thrombocytopenia in preventing platelet count recovery in thrombocytopenic patients with chronic liver disease. J. Gastroenterol. Hepatol..

[50] Xiao F., Tan S.L., Jing X., Meng F.K., Yu C., Li K., Cheng Z.-G., Yang J.-J., Zhu K.-S., Yu J. (2025). The application of avatrombopag in chronic liver disease patients with severe thrombocytopenia undergoing procedures in China: A prospective real-world cohort study. Int. J. Surg..

[51] Yang Z., Zhao Y., Chen H., Zhang H., Tan M., Li X., Tao L., Zhao H. (2025). Portal vein thrombosis in liver cirrhosis: A review of risk factors and predictive indicators. J. Clin. Transl. Hepatol..

[52] Nery F., Chevret S., Condat B., de Raucourt E., Boudaoud L., Rautou P.E., Plessier A., Roulot D., Chaffaut C., Bourcier V. (2015). Causes and consequences of portal vein thrombosis in 1243 patients with cirrhosis: Results of a longitudinal study. Hepatology.

[53] Huang X., Abougergi M.S., Sun C., Murphy D., Sondhi V., Chen B., Zheng X., Chen S., Wang Y. (2023). Incidence and outcomes of thromboembolic and bleeding events in patients with liver cirrhosis in the USA. Liver Int..

[54] Zanetto A., Pelizzaro F., Campello E., Bulato C., Balcar L., Gu W., Gavasso S., Saggiorato G., Zeuzem S., Russo F.P. (2023). Severity of systemic inflammation is the main predictor of ACLF and bleeding in individuals with acutely decompensated cirrhosis. J. Hepatol..

[55] Elvers F.L., Stamouli M., Adelmeijer J., Jeyanesan D., Bernal W., Maas C., Patel V.C., Lisman T. (2023). In vivo generation of thrombin in patients with liver disease without apparent evidence of activation of the intrinsic or extrinsic pathway of coagulation. J. Thromb. Haemost..

[56] Puente Á., Turón F., Martínez J., Fortea J.I., Guerra M.H., Alvarado E., Pons M., Magaz M., Llop E., Alvarez-Navascués C. (2025). Rivaroxaban to prevent complications of portal hypertension in cirrhosis: The CIRROXABAN study. J. Hepatol..

[57] Cox D. (2023). Sepsis—It is all about the platelets. Front. Immunol..

[58] El Otmani H., Frunt R., Smits S., Barendrecht A.D., de Maat S., Fijnheer R., Lenting P.J., Tersteeg C. (2024). Plasmin-cleaved von Willebrand factor as a biomarker for microvascular thrombosis. Blood.

[59] El Otmani H., Stamouli M., Adelmeijer J., Bernal W., Maas C., Patel V.C., Lisman T. (2025). Plasmin-mediated proteolysis of von Willebrand factor in patients with acute and chronic liver disease. Res. Pract. Thromb. Haemost..

[60] Pallotta D.P., Franceschini E., Boe M., Pratelli A., Monaco G., De Sinno A., Granito A. (2025). From thrombosis to transplantation: The role of anti-phospholipid antibodies in liver disease management. ILIVER.

[61] Basili S., Carnevale R., Nocella C., Bartimoccia S., Raparelli V., Talerico G., Stefanini L., Romiti G.F., Perticone F., Corazza G.R. (2019). Serum albumin is inversely associated with portal vein thrombosis in cirrhosis. Hepatol. Commun..

[62] Violi F., Corazza G.R., Caldwell S.H., Talerico G., Romiti G.F., Napoleone L., Perticone F., Bolondi L., Pietrangelo A., Vestri A.R. (2019). Incidence and recurrence of portal vein thrombosis in cirrhotic patients. Thromb. Haemost..

[63] Wang X., Huang J., Liu G., Xiang T., He Y., Wan S., Chen Z., Yang L., Luo X. (2025). Completely Occlusive Portal Vein Thrombosis as a Predictor of Mortality in Acute Variceal Bleeding. Liver Int..

[64] Lin C., Huang Y. (2025). Portal vein thrombosis and Esophageal-Gastric variceal bleeding in cirrhosis: Shared risk factors and causal relationship. BMC Gastroenterol..

[65] Tarar Z.I., Farooq U., Kamal F., Nawaz A., Saleem S., Ghous G., Basar O., Chela H.K., Tahan V., Daglilar E. (2023). Safety of anticoagulation use for treatment of portal vein thrombosis in liver cirrhosis and its effect on hospital-based outcomes: An insight from a US nationwide database. Postgrad. Med. J..

[66] Carlin S., Cuker A., Gatt A., Gendron N., Hernández-Gea V., Meijer K., Siegal D.M., Stanworth S., Lisman T., Roberts L.N. (2024). Anticoagulation for stroke prevention in atrial fibrillation and treatment of venous thromboembolism and portal vein thrombosis in cirrhosis: Guidance from the SSC of the ISTH. J. Thromb. Haemost..

[67] Zhou H., Wu M., Yu S., Xia H., Yu W., Huang K., Chen Y. (2023). Comparison of the efficacy and safety between rivaroxaban and dabigatran in the treatment of acute portal vein thrombosis in cirrhosis. BMC Gastroenterol..

[68] Baylo A., Cherniavskyi V., Reshotko D. (2023). Assessment of the efficiency and safety of anticoagulation therapy in patients with liver cirrhosis and atrial fibrillation. Clin. Exp. Hepatol..

[69] Niu C., Zhang J., Himal K., Zhu K., Zachary T., Verghese B., Jadhav N., Okolo P.I., Daglilar E., Kouides P. (2024). Impact of anticoagulation therapy on outcomes in patients with cirrhosis and portal vein thrombosis: A large-scale retrospective cohort study. Thromb. Res..

